# Welfare Work in Factories

**Published:** 1918-11-16

**Authors:** 


### Welfare Work in Factories.

THE CASE AGAINST THE CRECHE.

At a meeting of the Royal Sanitary Institute held at the College of Technology, Manchester, on October 18, Dr. Philip Boobbyer, Nottingham, who presided, said that if young women were to avoid shipwreck they must be provided with some respectable place of resort after their working hours. In many cases excellent provision on the lines of modern welfare work had been made in the factory, but little had been done in connection with the home life of the employees. This was a serious and fatal omission. With regard to the difficult question of the crèche, he said that the principle of the Maternity and Child Welfare Centres was to encourage mothers to stay at home, but crèches might encourage them to give up their responsibilities in the home and compete with men.

Many employers, said Professor J. Radcliffe, would have nothing to do with welfare work unless they were compelled by legislation, and many workers disregarded its advantages and often abused the facilities provided for their benefit. Careful observation in a small privately-owned factory convinced him that the adoption of welfare conditions placed no hardship of any kind on the owners, but increased the output of finished work by a body of contented and willing workers.

Miss Ethel Brown, a welfare worker at Rochdale, said that welfare work need not be regarded as philanthropic and sentimental. It was a sound business investment.

Sir Daniel McCabe said that the root of the trouble was low wages and improper feeding and housing. Much good would be done if employers set apart rooms where good meals could be had at reasonable prices.

Previous to the discussion, members of the Institute and other social workers paid a visit to the Tile and Pottery Works of Messrs. Pilkington and Co. at Clifton Junction. At these works the dangers of lead-glazing and those of other deleterious substances are understood to have been reduced, and the dust problem has been practically tackled.

